# Avian *Mycoplasma lipofaciens* Transmission to Veterinarian

**DOI:** 10.3201/eid1407.071703

**Published:** 2008-07

**Authors:** Michael Lierz, Andreas Jansen, Hafez M. Hafez

**Affiliations:** * Free University of Berlin, Berlin, Germany; † Robert Koch Institute, Berlin

**Keywords:** raptors, zoonoses, mycoplasmas, serology, turkeys, letter

**To the Editor:** *Mycoplasma* spp. are well-known pathogens in human and veterinary medicine. Mammals, especially primates and including humans, share similar or even identical *Mycoplasma* spp., which might be commensal or pathogenic (*1*). Additionally, sporadic infections of immunocompromised persons with *Mycoplasma* spp. that originated from domestic animals have been reported (*1*); susceptibility in this human population is increased (*2*,*3*). *M. phocicerebrale* is the only *Mycoplasma* pathogen of animals that regularly infects humans, causing a disease called seal fingers (*1*,*4*). However, we report a human infection with an avian *Mycoplasma* organism.

A clinical trial to investigate the capability of *M. lipofaciens* (strain ML64) (*5*) to spread horizontally between infected and noninfected turkey poults in an incubator demonstrated airborne transmission of the pathogen within 24 hours (*6*). During the trial, the veterinarian conducting the study, a 36-year-old man, was monitored for infection. Each day, 2 swabs were taken from both nostrils, starting from the day before the infected poults hatched (day 0) through day 7 after the poult hatching date. When handling eggs and poults, the veterinarian wore gloves but not a protective mask. Two days after the poults hatched (day 3), the veterinarian reported throat pain and a slight rhinitis, which indicated a respiratory disease. The next day only the rhinitis with minor nasal pain was present.

One nasal swab from each sampling day was used for *Mycoplasma* spp. culture (*5*). Isolated *Mycoplasma* organisms were subjected to an immunobinding assay (*6*) with antiserum against *M. lipofaciens*, *M. buteonis*, *M. falconis*, *M. gypis*, *M. gallisepticum*, *M. meleagridis*, *M. synoviae,* and *M. iowae,* selected because the veterinarian regularly handled these isolates. The second nasal swab from each sampling day was taken to detect *Mycoplasma* DNA by PCR (*5*). The samples for PCR testing were stored at –20°C and tested only when attempts to isolate *Mycoplasma* spp. failed.

Six weeks after the infection, a serum sample from the patient was examined for specific antibodies against *M. lipofaciens* by 3 different methods: modified immunobinding assay (*6*) (which used patient’s serum and peroxidase-conjugated goat-antihuman serum [STAR90P, Serotec Ltd, Oxford, UK]), a growth inhibition test, and antibody titers of serum dilutions. The first 2 methods used *M. lipofaciens* (strain ML64) from a turkey trial (*6*) and the reference strains of *M. buteonis* (Bb/T2g) and *M. falconis* (H/T1). For the third method, to determine the titer, 2-fold serial dilutions of the patient’s serum samples were prepared and incubated with a bacterial suspension (*M. lipofaciens* strain ML64) containing 3.2 × 10^2^ CFU. The antibody titer was based on the highest serum dilution capable of reducing 50% of the mean CFU count.

Before the infected poults hatched, attempts to isolate *Mycoplasma* organisms and demonstrate *Mycoplasma* DNA from the nasal swabs were unsuccessful. However, from the day of hatching (day 1) until 3 days later (day 4), *Mycoplasma* organisms were isolated from the nasal swabs and identified as *M. lipofaciens*. On day 5, only *Mycoplasma* DNA was demonstrated (Table). Specific antibodies against *M. lipofaciens* (strain ML64) were detected by using the immunobinding assay and growth inhibition test. Antibodies against other *Mycoplasma* spp. were not detected. Antibody titer against *M. lipofaciens* was 128.

**Table:** Timeline of natural infection of veterinarian with *Mycoplasma lipofaciens* (strain ML64) from infected poults*

Finding	Day							
	0	1†	2	3	4	5	6	7
Isolation	–	*M. l.*	*M. l.*	*M. l.*	*M. l.*	–	–	–
PCR result‡	–	ND	ND	ND	ND	+	–	–
Clinical signs	None	None	None	Throat pain, slight rhinitis	Slight rhinitis, nasal pain	None	None	None

*–, negative; *M.l., Mycoplasma lipofaciens* (identified by immunobinding assay); ND, not done; +, positive. †Hatching of infected poults and demonstration of aerosol transmission among poults. ‡Detection of mycoplasma DNA per Lierz et al. (*5*).

*M. lipofaciens* have been reported from a chicken, turkey, duck (*7*,*8*), and a raptor egg (*5*). Strain ML64 is highly pathogenic for chicken and turkey embryos and can be transmitted by air (*6*,*9*). The veterinarian handling the infected poults was free of nasal *Mycoplasma* organisms a day before contact. His infection occurred concurrent with demonstration of airborne transmission among poults. The isolation of *M. lipofaciens* from his nares for 4 days demonstrates the infectivity and reproductive capability of this *Mycoplasma* strain in humans: as a pure contaminant, isolation for several days would be unlikely. Christensen et al. (*10*) have reisolated different avian *Mycoplasma* strains (*M. gallisepticum*, *M. synoviae*, *M. iowae*) from a human nose, from 12 hours through 1 day after artificial infection, demonstrating differences between avian strain abilities to survive on human mucosa. *M. lipofaciens* invasiveness for humans is underscored by finding specific antibodies against this species 6 weeks after infection. Cross-reactivity to other *Mycoplasma* spp. cannot be excluded but seems unlikely.

This study suggests that *M. lipofaciens* (strain ML64) can be transmitted successfully to humans and may cause clinical symptoms; the study documents nonartificial human infection with an avian *Mycoplasma* sp. These findings should be considered especially for humans highly susceptible to *Mycoplasma* infections, including children and persons with congenital or acquired immunodeficiencies (*2*,*3*).
